# Preparation of Recombinant Human Collagen III Protein Hydrogels with Sustained Release of Extracellular Vesicles for Skin Wound Healing

**DOI:** 10.3390/ijms23116289

**Published:** 2022-06-03

**Authors:** Lanju Xu, Yufei Liu, Lizong Tang, Hui Xiao, Zhuo Yang, Shufang Wang

**Affiliations:** 1 Key Laboratory of Bioactive Materials for Ministry of Education, College of Medicine Sciences, Nankai University, Tianjin 300071, China; 1120190597@mail.nankai.edu.cn; 2 Key Laboratory of Bioactive Materials for Ministry of Education, College of Life Sciences, Nankai University, Tianjin 300071, China; 1120200555@mail.nankai.edu.cn (Y.L.); 2120201122@mail.nankai.edu.cn (L.T.); 2120201126@mail.nankai.edu.cn (H.X.)

**Keywords:** recombinant human collagen Ⅲ protein, extracellular vesicles, hydrogels, wound healing

## Abstract

Existing treatment methods encounter difficulties in effectively promoting skin wound healing, making this a serious challenge for clinical treatment. Extracellular vesicles (EVs) secreted by stem cells have been proven to contribute to the regeneration and repair of wound tissue, but they cannot be targeted and sustained, which seriously limits their current therapeutic potential. The recombinant human collagen III protein (rhCol III) has the advantages of good water solubility, an absence of hidden viral dangers, a low rejection rate and a stable production process. In order to achieve a site-specific sustained release of EVs, we prepared a rhCol III hydrogel by cross-linking with transglutaminase (TGase) from *Streptomyces mobaraensis*, which has a uniform pore size and good biocompatibility. The release profile of the rhCol III-EVs hydrogel confirmed that the rhCol III hydrogel could slowly release EVs into the external environment. Herein, the rhCol III-EVs hydrogel effectively promoted macrophage changing from type M1 to type M2, the migration ability of L929 cells and the angiogenesis of human umbilical vein endothelial cells (HUVECs). Furthermore, the rhCol III-EVs hydrogel is shown to promote wound healing by inhibiting the inflammatory response and promoting cell proliferation and angiogenesis in a diabetic rat skin injury model. The reported results indicate that the rhCol III-EVs hydrogel could be used as a new biological material for EV delivery, and has a significant application value in skin wound healing.

## 1. Introduction

Skin is an important barrier for the human body, but it is often damaged, resulting in refractory wounds [1]. The main barriers to wound healing include diabetes, vascular insufficiency and local pressure changes [2]. Diabetes is one of the most common public health problems in the world [3]. Diabetic foot ulcers (DFUs) are a common and severe chronic complication among diabetic patients, and the main cause of non-traumatic amputations [4,5]. At present, the routine clinical treatments for skin wounds mainly include surgical debridement, negative pressure therapy and repeated changes in wound dressings. However, due to the disfunction of cells within the wound during the healing process, the therapeutic effects of these treatments are still unsatisfactory [6].

The ability of mesenchymal stem cells (MSCs) to differentiate into different skin cells, as well as their unique anti-inflammatory properties within wounds, have attracted the attention of more and more scholars [7]. At present, bone marrow mesenchymal stem cells (BMSCs), adipose mesenchymal stem cells (ADSCs), dental pulp mesenchymal stem cells (DPSCs) and human umbilical cord mesenchymal stem cells (hUC-MSCs) are widely used. Compared with other types of stem cells, hUC-MSCs have the advantages of being available from a wide range of sources, possessing strong expansion abilities and exhibiting low immunogenicity [8]. However, the direct use of MSC transplantation treatments still possesses some limitations, related to abnormal cell phenotypes, low homing efficiency, cell differentiation and proliferation changes [9]. The value of MSCs in clinical therapy is attributed to their paracrine effects. In addition, EVs secreted by MSCs through paracrine regulation, also exert mainly therapeutic effects [10,11,12].

EVs have the same effect as MSCs and are easier to store and transport; they can also cross biological barriers and mediate biologically active molecules to transmit information among cells [10,13,14]. Furthermore, due to their regulation of biological behaviors such as cellular immunity, proliferation, migration and angiogenesis, EVs offer wide therapeutic effects in the context of wound repair [15,16]. Intravenous injection is a common route for the administration of EVs, but EVs can be easily cleared via blood circulation and subsequently accumulate in the liver, spleen and lungs. Following intravenous injection, EVs isolated from mouse melanoma cells were rapidly eliminated from circulation, with a half-life of approximately 2 min [17]. Blood clearance and the accumulation of EVs at off-target sites seriously affect their efficacy and safety as therapies [18].

This problem of rapid EV removal could be effectively solved via the development of hydrogel materials as a novel strategy for EV delivery. Hydrogels have three-dimensional polymer structures, which are conducive to encapsulating bioactive molecules and contribute to wound healing in humid environments [19,20]. Collagen hydrogels have been well studied and can be applied in tissue engineering, especially in the field of wound repair [21,22,23]. Collagen is the most commonly used biomedical material in this context, possessing good biocompatibility and favorable biomimetic and hemostatic properties. Currently, commercial collagen is mainly derived from animal tissue, and suffers from poor solubility, batch-to-batch purity variation and pathogenic contamination [24]. Recombinant collagen is a novel biomaterial manufactured by synthetic biology techniques and is a candidate material for various medical applications [25]. Type III collagen is a major component of the extracellular matrix in internal organs and skin in adults [26]. The recombinant human collagen III protein (rhCol III) is the product of a re-optimized design gene sequence based on the characteristics and main functional domains of human collagen. In addition, rhCol III could promote skin repair and regeneration [27]. In the present study, a novel type of hydrogel material was prepared by using rhCol III as the raw material to achieve the sustained release of EVs in skin wounds.

In summary, we loaded the rhCol III hydrogel with EVs secreted by hUC-MSCs in order to obtain a hydrogel material with a sustained-release function. Herein, we explore its effects on the biological functions of wound-healing-related cells and its therapeutic effect on the repair of full-thickness skin wounds in diabetic rats. Overall, the rhCol III-EVs hydrogel was designed and synthesized to facilitate the sustained release of EVs, and is expected to provide an effective new therapy for skin wound healing.

## 2. Results

### 2.1. Isolation and Identification of hUC-MSCs-Derived EVs

**Figure 1:**
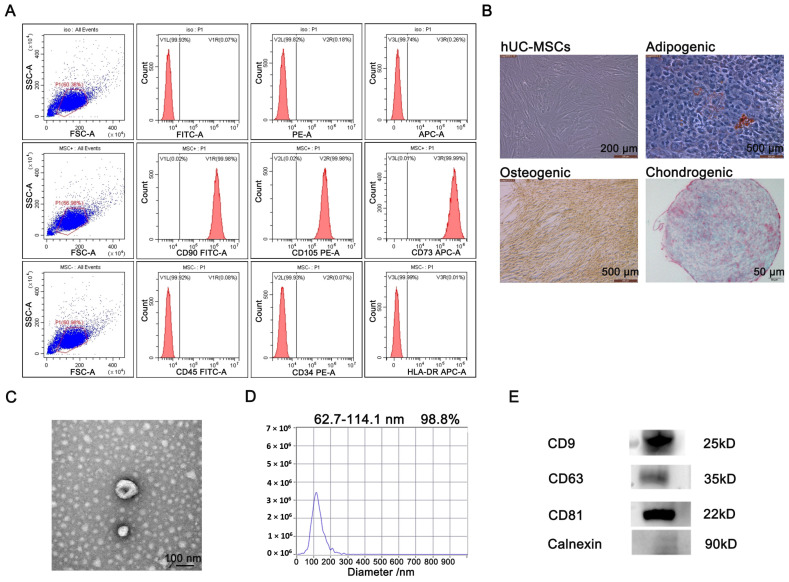
Identification and characterization of hUC-MSCs and secreted EVs. (**A**) Identification of surface marker proteins of hUC-MSCs by flow cytometry. (**B**) The differentiation ability of hUC-MSCs was evaluated by cell staining. (**C**) The morphological characteristics of the EVs were observed by TEM. (**D**) The particle size distribution range of the EVs was analyzed by NTA. (**E**) The surface markers of EVs were analyzed by Western blot.

hUC-MSCs were identified via flow cytometry analysis and cell staining experiments. The flow cytometry analysis confirmed that hUC-MSCs were positive for CD90 (99.98%), CD105 (99.98%) and CD73 (99.99%), and negative for CD45 (0.08%), CD34 (0.07%) and HLA-DR (0.01%) (Figure 1A). The bioavailability of hUC-MSCs was verified via a staining experiment to determine cell differentiation. As shown in Figure 1B, hUC-MSCs could induce differentiation into adipogenic, osteogenic and chondrogenic phenotypes. The above results indicate that the cells exhibited biological differentiation, which is consistent with the biological characteristics of hUC-MSCs. The EVs collected from the culture supernatant of hUC-MSCs were extracted and identified. Using transmission electron microscopy (TEM), we found that hUC-MSCs-EVs were double-layered vesicles with a shape similar to a cup or sphere (Figure 1C). NanoSight Analysis (NTA) determined that the mean particle size of hUC-MSCs-EVs was 62.7–114.1 nm and 98.8% of all particles were within this size range (Figure 1D). Additionally, hUC-MSCs-EVs significantly expressed the EV-labeled proteins CD9, CD63 and CD81, while the negative control Calnexin protein was not expressed (Figure 1E). Thus, hUC-MSCs-EVs were successfully separated.

### 2.2. Preparation and Characterization of the rhCol III Hydrogel

**Figure 2:**
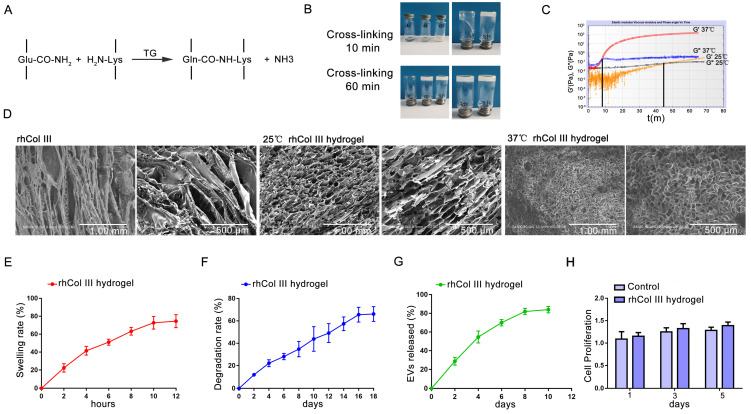
Preparation of the rhCol III hydrogel. (**A**) Schematic diagram of the reaction of the rhCol III hydrogel preparation. (**B**) TGase crosslinked to the rhCol III hydrogel. (**C**) Rheological experiments of the rhCol III hydrogel. (**D**) SEM image of the rhCol III hydrogel. (**E**) Swelling rate of the rhCol III hydrogel. (**F**) Degradation rate of the rhCol III hydrogel. (**G**) Sustained release of EVs in the rhCol III hydrogel. (**H**) Effect of the rhCol III hydrogel extract on cell proliferation (*n* = 3).

TGase is a kind of non-immunogenic cross-linking agent and has low cytotoxicity compared with the common cross-linking agents Genipin and glutaraldehyde (GA) [28]. TGase could catalyze the reaction of the g-acyl group of glutamine (Glu) and the ε-amine group of lysine (Lys) bonding to cross-link protein molecules. The rhCol III polypeptide chain contains 4.30% Glu and 2.90% Lys. Therefore, the TGase was used to crosslink the rhCol III hydrogel (Figure 2A). As shown in Figure 2B, under the reaction conditions of 25 °C or 37 °C, the fluidity of the reaction system reduced, confirming that the cross-linking reaction had occurred. A rheological experiment was used to detect the gelling time of the rhCol III hydrogel, G′ and G″. The gelation time of the rhCol III hydrogel was 48 min at 25 °C and 7 min at 37 °C (Figure 2C). After lyophilization of the rhCol III hydrogel, its pore size was examined by SEM. The result of the SEM showed that the rhCol III hydrogel obtained by crosslinking at 37 °C had an interconnected homogeneous 3D porous microstructure (Figure 2D). According to the above results, the rhCol III hydrogel prepared at 37 °C had a shorter gelation time. Importantly, the rhCol III hydrogel gelled at 37 °C had a more uniform pore size and a 3D network-like structure. Therefore, in order to efficiently obtain hydrogels with a uniform pore size, the subsequent preparation of the rhCol III hydrogel was conducted at 37 °C. The swelling properties of hydrogels enable them to absorb wound exudate, thereby maintaining a clean and moist environment for the wound. After 12 h, the swelling rate of the rhCol III hydrogel was about 75% (Figure 2E). Hydrogels can also provide adhesion and proliferation sites for cells in tissue engineering, and their degradation rate should match the generation of new tissue [29]. After 14 days, the hydrogel was degraded by about 60% (Figure 2F). The membrane surface of EVs has g-acyl group and ε-amine group [30]. Therefore, TGase could enable EVs to be loaded onto the rhCol III hydrogel, thereby safely and effectively avoiding the sudden release of EVs. The EVs in the rhCol III-EVs hydrogel were released slowly, reaching about 80% release at 10 days (Figure 2G). At 1, 3 and 5 days, there was no statistically significant difference between the Control group and the rhCol III hydrogel group in terms of cell proliferation (Figure 2H). Taken together, these findings indicate that this novel hydrogel material could be used as a delivery medium for EVs and has good biocompatibility.

### 2.3. Cellular Inflammation and Migration Regulation

**Figure 3:**
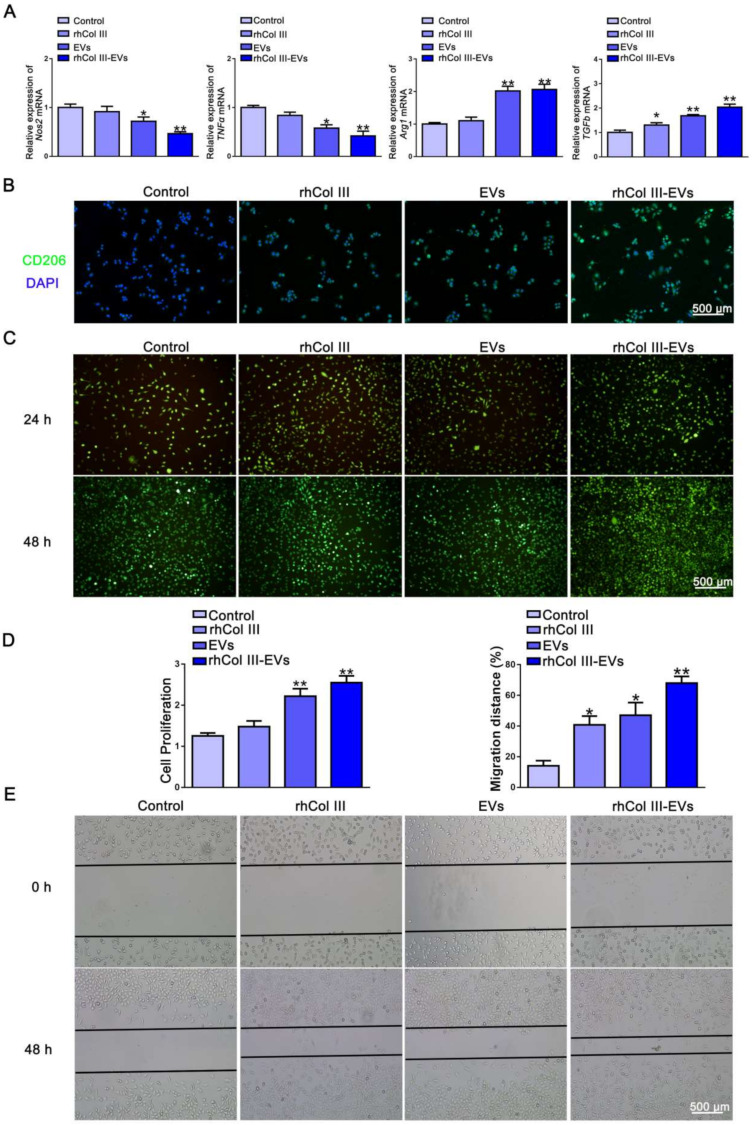
The differentiation of RAW264.7 cells and the migration of L929 cells. (**A**) QRT-PCR to detect the expression of M1 and M2 molecular markers in RAW264.7 cells. (**B**) Immunofluorescence (green) showing the number of CD206-positive cells in RAW264.7 cells (*n* = 3). (**C**) Live/dead analysis of L929 cells in different groups (*n* = 3). (**D**) Proliferation of L929 cells in different groups (*n* = 3, * *p*-value < 0.05 vs. Control group, ** *p*-value < 0.01 vs. Control group). (**E**) Scratch test detected the migration ability of L929 cells (*n* = 3, * *p*-value < 0.05 vs. Control group, ** *p*-value < 0.01 vs. Control group).

The effects of gradient concentrations of EVs on the proliferation of the HUVECs, L929 cells and RAW264.7 cells were examined. The results showed that EVs at concentrations of 20 μg/mL, 40 μg/mL and 60 μg/mL could all promote the proliferation of HUVECs, L929 cells and RAW264.7 cells. As the concentration of EVs increased, the HUVECs, L929 cells and RAW264.7 cells showed a higher proliferation ability (Figure S1). The EVs promoted cell proliferation in a concentration-dependent manner. Since 60 μg/mL EVs exhibited a more obvious effect in promoting cell proliferation, this concentration was selected for subsequent cell-function testing. Long-term inflammation plays a major role in defective wound healing and the development of chronic wounds [31]. M1 phenotype macrophages mainly play a pro-inflammatory role. M2 phenotype macrophages mainly function as anti-inflammatories and in promoting tissue repair. The RAW264.7 cells can regulate the process of inflammation by adjusting the polarization of their M1 and M2 phenotypes. *Nos2* and *TNFα* were selected as the related genes of M1-type macrophages. *Arg1* and *TGFb* were selected as the related genes of M2-type macrophages. The primers used are shown in Table 1. The results showed that the composite hydrogel significantly reduced the mRNA levels of the pro-inflammatory genes *Nos2* and *TNFα*, and significantly increased mRNA levels of the anti-inflammatory genes *Arg1* and *TGFb* (Figure 3A). To further verify the results, immunofluorescence staining of CD206 was performed in this study. CD206 is a specific molecular marker for M2-type macrophages. The number of CD206-positive cells in the rhCol III-EVs group was greater than that in the Control group, rhCol III group and EVs group (Figure 3B). The above results indicate that the composite rhCol III-EVs hydrogel promoted M2-type transformations of RAW264.7 cells.

The proliferation and migration abilities of fibroblasts are very important for new granulation tissue and wound repair. After 24 h and 48 h, the L929 cells in the rhCol III groups, EVs groups and rhCol III-EVs groups survived well, and no dead cells were observed (Figure 3C). We further explored the effect of the composite hydrogel material on cell proliferation and migration. The rhCol III, EVs and rhCol III-EVs all promoted the proliferation ability of L929 cells (Figure 3D). The scratch results showed that the cell migration rate was 14% in the Control group and 70% in the rhCol III-EVs group (Figure 3E). The above results demonstrate that the rhCol III-EVs hydrogel promoted the proliferation and migration of L929 cells.

### 2.4. Angiogenesis Regulation

**Figure 4:**
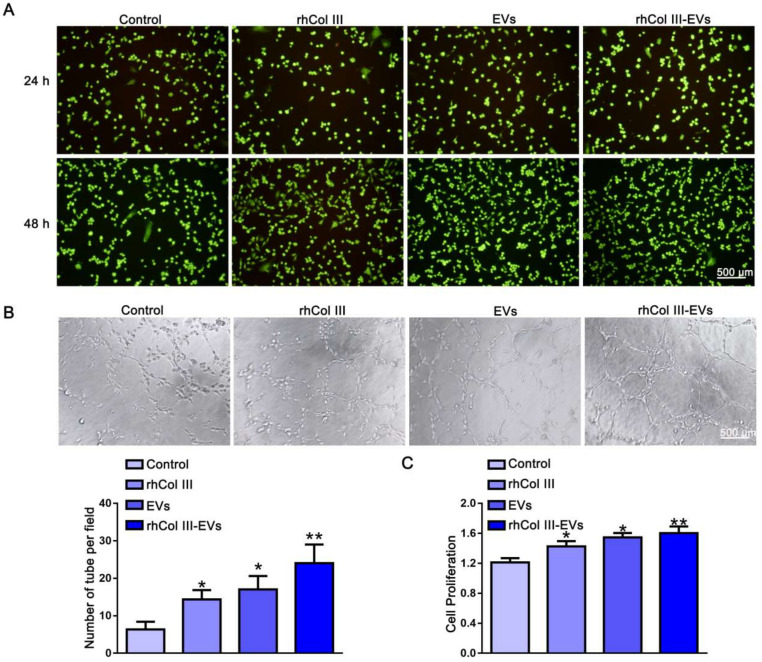
The effect of rhCol III-EVs on the angiogenesis of HUVECs. (**A**) Live/dead analysis of HUVECs in different groups (*n* = 3). (**B**) Angiogenesis of HUVECs in different groups (*n* = 3, * *p*-value < 0.05 vs. Control group, ** *p*-value < 0.01 vs. Control group). (**C**) Proliferation of HUVECs in different groups (*n* = 3, * *p*-value < 0.05 vs. Control group, ** *p*-value < 0.01 vs. Control group).

Live/Dead staining confirmed that the rhCol III-EVs had good biocompatibility with the HUVECs (Figure 4A). A HUVECs tube generation experiment was used to evaluate the angiogenesis ability of the HUVECs. Compared with the Control, rhCol III and EVs, the rhCol III-EVs significantly promoted the level of HUVECs angiogenesis (Figure 4B). The rhCol III-EVs group showed greater promotion of cell proliferation (Figure 4C). Based on the above results, the rhCol III-EVs promoted the angiogenesis ability of the HUVECs.

### 2.5. Wound Healing in Diabetic Rats

**Figure 5:**
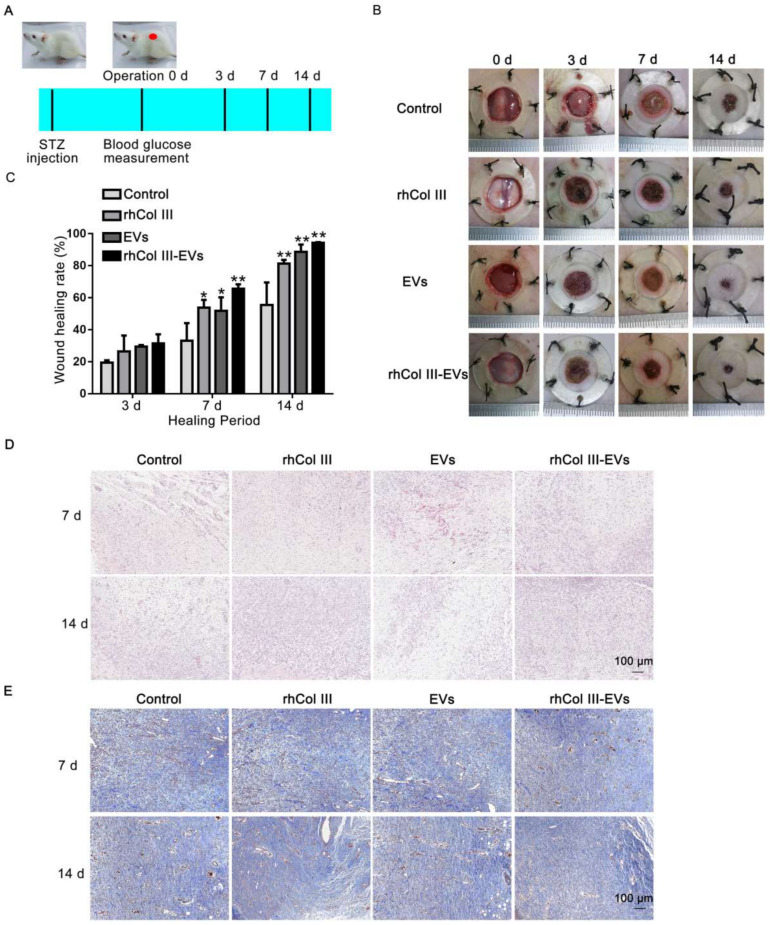
The effect of experimental group on wound healing in vivo. (**A**) Schematic diagram of diabetic rat wound model. (**B**) Representative wound images from each group at 0, 3, 7 and 14 days. (**C**) Wound healing rate of each group at different times (*n* = 3, * *p*-value < 0.05 vs. Control group, ** *p*-value < 0.01 vs. Control group). (**D**) HE analysis of wound tissue in each group at 7 and 14 days. (**E**) Masson analysis of wound tissue in each group at 7 and 14 days.

To evaluate the therapeutic effect of the rhCol III-EVs hydrogel, its regulation on the healing of back skin wounds in diabetic rats was examined (Figure 5A). The rhCol III hydrogel group, EVs group and rhCol III-EVs hydrogel group all exhibited greatly improved wound repair effects and promotion of wound healing. It is important to note that the rhCol III-EVs hydrogel group exhibited the greatest effect (Figure 5B). At day 14, the wound healing rate in the rhCol III-EVs hydrogel group was 94% (Figure 5C).

Skin granulation in the wound tissue was more pronounced in the rhCol III-EVs hydrogel group (Figure 5D). Via Masson staining, the rhCol III-EVs hydrogel group was found to exhibit more collagen deposition and ordered fibers (Figure 5E). The above results confirm that the designed rhCol III-EVs composite hydrogel could promote wound tissue regeneration and collagen deposition in diabetic wounds.

### 2.6. Cellular Inflammation and Proliferation Changes in Wound Repair

**Figure 6:**
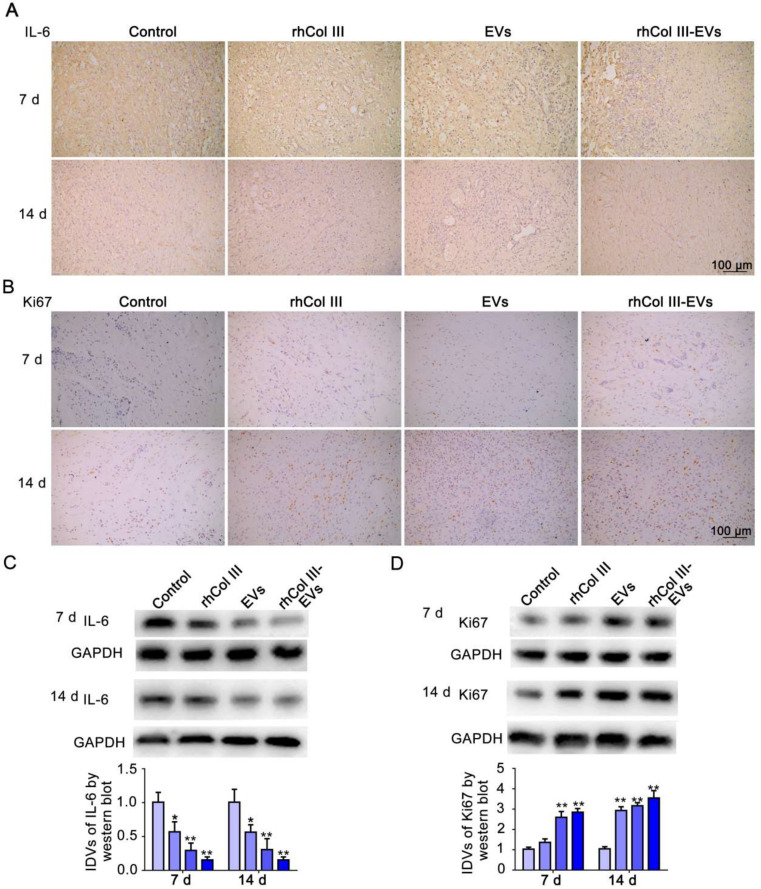
Expression analysis of IL-6 and Ki67 in wound tissue. (**A**) Immunohistochemistry images for IL-6 at 7 and 14 days. (**B**) Immunohistochemistry images for Ki67 at 7 and 14 days. (**C**) Western blot for IL-6 at 7 and 14 days (*n* = 3, * *p*-value < 0.05 vs. Control group, ** *p*-value < 0.01 vs. Control group). (**D**) Western blot for Ki67 at 7 and 14 days (*n* = 3, * *p*-value < 0.05 vs. Control group, ** *p*-value < 0.01 vs. Control group).

IL-6 is commonly used as a marker of inflammatory response in diabetic wound tissue [32]. The immunohistochemical analysis indicated that the expression level of IL-6 in the rhCol III-EVs hydrogel group was significantly reduced (Figure 6A). This result demonstrates that the rhCol III-EVs hydrogel inhibited the inflammatory reaction in the skin wound tissue. Ki67 is an important molecular indicator to evaluate cell proliferation [33]. It could be observed that the ratio of Ki67-positive cells in the rhCol III-EVs hydrogel group was significantly increased (Figure 6B). At 7 and 14 days, the expression of IL-6 in the rhCol III-EVs hydrogel group was significantly decreased and Ki67 was significantly increased, as determined by Western blot (Figure 6C–D). These results indicate that the rhCol III-EVs hydrogel reduced the inflammatory response and enhanced the proliferation ability of cells in wound tissues.

### 2.7. Angiogenesis of Skin Wound Tissue

**Figure 7:**
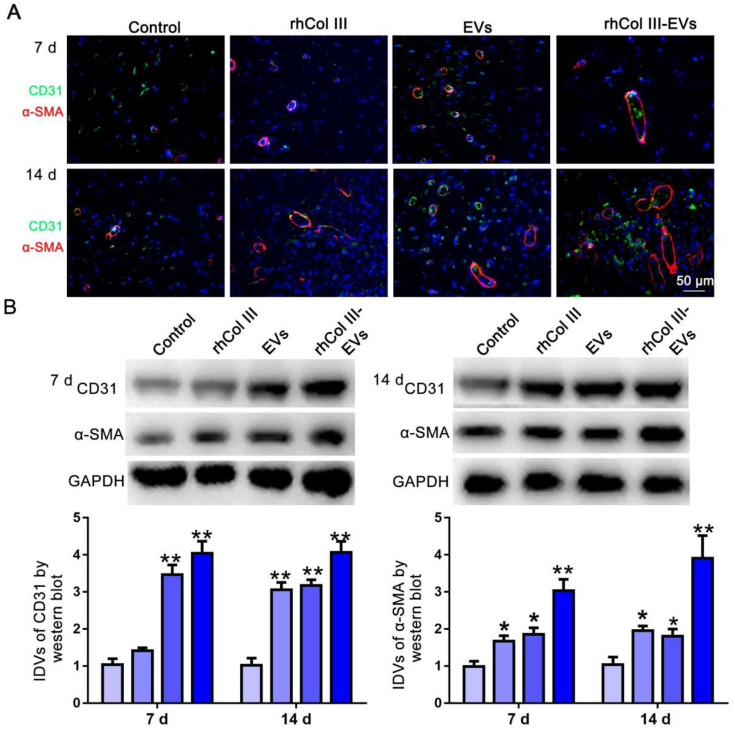
Expression analysis of CD31 and α-SMA in wound tissue. (**A**) Immunofluorescence images for CD31 and α-SMA at 7 and 14 days. (**B**) Western blot for CD31 and α-SMA at 7 and 14 days (*n* = 3, * *p* < 0.05, ** *p* < 0.01).

CD31 is a transmembrane protein expressed in early angiogenesis, indicating neovascularization, while α-SMA is a cytoplasmic protein expressed in later angiogenesis, indicating the maturation of vascular smooth muscle cells [34]. The vascular network plays a key role in the repair of full-thickness skin due to its function of transporting nutrients, oxygen and immune cells into the wound area [26]. CD31/α-SMA immunofluorescence was performed on the wound tissues at 7 and 14 days. As shown in Figure 7A, in the rhCol III-EVs hydrogel group, the fluorescence intensity of CD31 and α-SMA at 7 and 14 days was obvious and the distribution area was large. It could also be observed that the expressions of CD31 and α-SMA were significantly increased at 7 and 14 days (Figure 7B). These results confirm that the rhCol III-EVs hydrogel had an obvious promoting effect on angiogenesis after injury.

## 3. Discussion

EVs are lipid bilayer vesicles derived from cells, serum or other biological fluids, and are composed of bioactive molecules, including mRNA, miRNA, DNA, lipids, proteins and metabolites, which are involved in biological signal transduction between cells, and, due to their ability to modulate various biological processes, they are potential new mediators for disease treatments, diagnostic biomarkers and drug delivery systems [35]. MSC-derived EVs (MSC-EVs) can deliver a variety of molecules including lipids, proteins and nucleic acids to target cells and play an important regulatory role [36]. EVs isolated from umbilical cord blood (CB) and adipose tissue (AT)-derived MSCs contain cardioprotective and tumor-suppressive miRNAs, such as miR-22-3p, miR-26a-5p and miR-16-5p, etc. [37]. EVs have beneficial effects on cells involved in skin wound healing by affecting the processes of cell proliferation and vascularization [38]. Since direct injection of EVs into wounds results in the loss and escape of EVs, we designed and synthesized a rhCol III hydrogel to achieve local sustained release of EVs. The release profile of the embedded EVs confirmed that the rhCol III-EVs hydrogel achieved local sustained release of EVs. Importantly, in the repair of skin wounds, by realizing the functions of rhCol III and EVs, this rhCol III-EVs hydrogel could possess excellent therapeutic advantages compared with the application of the rhCol III hydrogel or EVs alone.

The wound healing process is divided into hemostatic, inflammatory, proliferative and remodeling phases, all of which must occur in an appropriate sequence and timeframe [39]. The phenotypic transition of macrophage polarization during inflammation is an important step in wound healing, and, when tissue begins to repair in acute wounds, the entire macrophage population shifts to the M2 phenotype with anti-inflammatory and regenerative effects [40]. Therefore the rhCol III-EVs hydrogel is able to modulate the inflammatory response, which is crucial for its clinical application. We observed that the mRNA levels of *Arg1* and *TGFb*, which are the marker proteins of M2-type macrophages, were significantly increased in the rhCol III-EVs group. In this study, we also demonstrated that the number of CD206-positive cells increased in the rhCol III-EVs hydrogel group, as determined by immunofluorescence experiments. In the above experiments, the rhCol III-EVs hydrogel could better regulate macrophages to transform into the anti-inflammatory phenotype. Multiple studies have shown that MSC-EVs are less immunogenic and inhibit the development and progression of experimental diabetes in animal models by modulating the M1/M2 balance [41]. MSC-EVs express several adhesion molecules (CD29, CD44 and CD73) that allow them to home to injured and inflamed tissues. In a mouse model of acute kidney injury (AKI), MSC-EVs mainly accumulated in the inflamed kidney, whereas in a model of intracerebral hemorrhage, they were detected in the injured brain [42]. It has been reported that MSC-EVs promote differentiation into M2-type macrophages through the miR-223/pKNOX1 pathway to exert anti-inflammatory properties and enhance skin wound healing [43]. During the proliferative phase, skin fibroblasts with improved proliferation and migration ability could stimulate the production of the extracellular matrix, which is beneficial to wound healing [44]. Our results indicate that the rhCol III-EVs promoted L929 cells’ proliferation and migration ability compared with either rhCol III hydrogel or EVs alone. In addition to inflammation and wound cell migration, nutrient transport at the wound site is also an important factor affecting wound repair. In our study, loading EVs in rhCol III hydrogels better promoted the proliferation and angiogenesis of the HUVECs. This result indicates that the rhCol III-EVs hydrogel could enhance nutrient delivery to the wound site. Previous studies have shown that the enrichment of VEGF-A in EVs can enhance the angiogenic activity of the injured spinal cord, and accelerate microvascular regeneration in mice with spinal cord injuries [45]. By examining the effect of the rhCol III-EVs hydrogel on the biological functions of skin-wound-related cells, this study confirmed that the rhCol III-EVs hydrogel played an important role in regulating cellular inflammation, migration and vascularization in skin wounds.

A full-thickness skin defect model in diabetic rats could mimic the wound healing process in diabetic patients. Skin wounds were made on backs of diabetic rats to explore the reparative effects of the rhCol III-EVs hydrogel. We confirmed that the rhCol III-EVs hydrogel promoted wound healing, granulation regeneration and collagen deposition in the wound compare with either rhCol III hydrogel or EVs alone. Furthermore, in the rhCol III-EVs hydrogel group, the inflammatory factor IL-6 was more significantly decreased, while the cell proliferation factor Ki67 and the vascularization factors CD31 and α-SMA were more significantly increased, confirming that the rhCol III-EVs hydrogel accelerated the wound healing process by affecting the inflammatory response, proliferation and vascularization of cells throughout the whole wound healing process. Based on the results in vitro and in vivo, the rhCol III-EVs hydrogel could reduce the inflammatory response of wounds and promote the migration and vascularization of wound tissue cells, and is conducive to the maintenance of wound nutrition and accelerated wound tissue regeneration.

## 4. Materials and Methods

### 4.1. Materials

The rhCol III (70 kDa) was provided by Hebei NACOL Biotechnology Co., Ltd. (Shijiazhuang, Hebei, China). TGase and Tris-HCl reagents were purchased from Solarbio Biotechnology Co., Ltd. (Beijing, China). Dulbecco’s modified Eagle’s medium (DMEM), 1640 medium and fetal bovine serum (FBS) were purchased from Gibco (New York, NY, USA). ECM complete medium was purchased from Sciencell (Carlsbad, CA, USA). Cell counting kit (CCK-8) was purchased from Seven Biotechnology Co., Ltd. (Beijing, China). Live/Dead staining kit was purchased from Yuanye Biotechnology Co., Ltd. (Shanghai, China). MonScript™ RTIII All-in-One Mix with dsDNase and MonAmp™ ChemoHS qPCR Mix were purchased from Monad Biotechnology Co., Ltd. (Suzhou, Jiangsu, China). RIPA lysate and ECL Luminescence Kit were purchased from Beyotime Biotechnology Co., Ltd. (Shanghai, China). Matrigel was purchased from Corning (New York, NY, USA). The primer was purchased from Sangon Biotechnology Co., Ltd. (Shanghai, China). The antibody brands and product numbers used are as follows: CD90 (Biolegend, 328108), CD105 (Biolegend, 323206), CD73 (Biolegend, 344006), CD34 (Biolegend, 343505), CD45 (Biolegend, 304006), HLA-DR (Biolegend, 307609), CD9 (Proteintech, 20597-1-AP), CD63 (Proteintech, 25682-1-AP), CD81 (Proteintech, 66866-1-Ig), Calnexin (Proteintech, 10427-2-AP), CD206 (Proteintech, 18704-1-AP), CD31 (Santa, sc-376764), IL-6 (Affinity, DF6087), α-SMA (Affinity, AF1032) and GAPDH (Proteintech, 60004-1-Ig).

### 4.2. Cell Culture

The mouse peritoneal macrophage cell line (RAW264.7 cells), the mouse skin fibroblasts cell line (L929 cells) and the human umbilical vein endothelial cells (HUVECs) were all gifts from Professor Shufang Wang, School of Life Sciences, Nankai University. The HUVECs were purchased from ScienceCell Research Laboratories, Lot number 28181, CA number 0004054. The RAW264.7 cells were cultured in DMEM medium with 10% FBS; L929 cells were cultured in 1640 medium with 10% FBS; and HUVECs were cultured in ECM complete medium.

### 4.3. Identification of hUC-MSCs

Identification of hUC-MSCs was performed as previously described [46]. hUC-MSCs were trypsinized for 4 min, washed with PBS without calcium and magnesium and blocked by adding 10% normal goat serum. After blocking, the cells were co-incubated with antibodies and resuspended in 10% normal goat serum for flow cytometry.

### 4.4. Extraction of EVs

The hUC-MSC culture supernatant was centrifuged at 300 *g* for 10 min at 4 °C. The supernatant was absorbed and centrifuged at 2000 *g* for 10 min. Then the aspirated supernatant was centrifuged at 10,000 *g* for 70 min. After the supernatant was centrifuged again at 12,000 *g* for 90 min, the EVs were obtained. The EVs were dissolved in PBS and stored at −80 °C.

### 4.5. Identification of EVs

The TEM detection for EVs was conducted as follows. After washing the copper mesh with PBS and drying, the EVs were dropped on copper grids, stained with uranyl acetate staining solution and photographed using a Hitachi-HT 7800 transmission electron microscope.

The NTA detection for EVs was conducted as follows. The sample wells were washed with deionized water and PBS buffer. The EVs were then diluted with PBS and detected using a ZetaView instrument.

The Western blot for EVs was conducted as follows. Protein quantification of EVs was performed using the Bicinchonininc Acid (BCA) method. A quantity of 10 μg protein of EVs was subjected to SDS-PAGE electrophoresis. After electrophoresis, transfer to PVDF membrane and incubation with antibodies were performed. After incubation with primary and secondary antibodies, ECL luminescence visualization was performed.

### 4.6. RhCol III Hydrogel Preparation

Here, the TGase cross-linking method was applied to the synthesis of the rhCol III hydrogel. Briefly, the 2.5% rhCol III was dissolved in Tris-HCl buffer at pH 8.0. Then, under slow stirring, TGase powder with a reaction concentration of 1000 U/g was added. After the TGase was fully dissolved, the reaction was carried out at 25 °C or 37 °C.

### 4.7. Scanning Electron Microscopy (SEM)

The lyophilized rhCol III hydrogel was sprayed with gold and placed in the SEM sample stage. Pore size of the rhCol III hydrogel was recorded by SEM.

### 4.8. Swelling Rate

The rhCol III hydrogel was immersed in PBS at 37 °C. The initial mass was taken before immersion and denoted as M_0_. At each time point, the rhCol III hydrogel was taken to remove the PBS on the surface. The weight was taken and denoted as M_1_. The swelling rate of the rhCol III hydrogel was (M_1_ − M_0_)/M_0_ × 100%.

### 4.9. Degradation Rate

After swelling for 24 h, the rhCol III hydrogel was immersed in PBS at 37 °C. The swelled mass was weighed and denoted as M_0_. At each time point, the hydrogel was taken to remove water from the surface, and the mass at this time was weighed and denoted as M_1_. The degradation rate of the rhCol III hydrogel was (M_0_ − M_1_)/M_0_ × 100%.

### 4.10. Rheological Test

The storage modulus (G′), loss modulus (G″) and gel point of the rhCol III hydrogel during gelation were scanned according to single frequency oscillation time. A quantity of 1 mL of the rhCol III hydrogel reaction solution was dropped into the test fixture, and the detection parameters were as follows: frequency 1 Hz; strain 1%; and temperature 25 °C or 37 °C. During the gel formation process of the rhCol III hydrogel, the curve of the gel point time, and the changing law between G′ and G ″ were recorded.

### 4.11. EVs Sustained Release Detection

Sustained release detection of EVs was performed as previously described [6]. The BCA method was used to detect the sustained release rate of EVs.

### 4.12. Cell Proliferation

The cells were plated in a 96-well plate at a density of 3000 cells per well. After 24 h, the rhCol III hydrogel extract, EVs and the mixture of rhCol III hydrogel extract and EVs were added to the well plate, and the culture was continued for 48 h. The CCK-8 reagent was added to each well, and the OD value was read at a wavelength of 450 nm after 1.5 h of reaction. The groups were as follows: Control group, rhCol III (rhCol III hydrogel extract) group, EVs group and rhCol III-EVs (rhCol III hydrogel extract+EVs) group.

### 4.13. Live/Dead Staining

The cells were plated in a 24-well plate. After 24 h, the rhCol III hydrogel extract, EVs and the mixture of rhCol III hydrogel extract and EVs were added to the well plate for further incubation. After 24 h and 48 h, the plate was taken out and washed with PBS. Cells were stained for 15 min with a working solution of propidium iodide (PI), calcein AM (AM) and PBS solution. PI stain stains dead cells and presents red fluorescence, whereas AM stain stains live cells and presents green fluorescence.

### 4.14. Quantitative Real-Time PCR (qRT-PCR)

Reverse transcription and amplification of RNA were performed according to the procedures of MonScript™ RTIII All-in-One Mix with dsDNase and MonAmp™ ChemoHS qPCR Mix kits. *GAPDH* was selected as the internal reference, and the relative expression of the gene was calculated by the 2^−^^△△Ct^ method. The expression levels of nitric oxide synthase 2 (*Nos2*), tumor necrotic factor-alpha (*TNFα*), arginase 1 (*Arg1*) and transforming growth factor beta (*TGFb*) were detected. The primer sequence used was as follows:

### 4.15. CD206 Immunofluorescence

Coverslips were placed in six-well plates, surfaced with gelatin and incubated for 30 min. The RAW264.7 cells were seeded in six-well plates. After 24 h, the rhCol III hydrogel extract, EVs and the mixture of rhCol III hydrogel extract and EVs were added to the plate, and the incubation was continued for 48 h. The slides were fixed with 4% paraformaldehyde for 30 min and blocked with 5% BSA for 2 h. The primary antibody was kept at 4 °C overnight, and the secondary antibody incubated for 2 h at room temperature. Nuclei were stained with DAPI staining solution in the dark for 5 min. Finally, the slides were mounted with anti-fluorescence quenching glycerol.

### 4.16. Angiogenesis Detection of HUVECs

The Matrigel was pipetted into a 48-well plate and incubated for 2 h. The cell density of HUVECs was adjusted to 10 × 10^4^/mL and 300 μL was added to the well plate. Then, the well plate was incubated for 6 h and pictures were taken to detect the blood vessel concentration of HUVECs.

### 4.17. Skin Wound Healing Model in Rats

In this study, all animal procedures were approved by the Animal Care Committee and followed the regulations of the Administration of Affairs Concerning Experimental Animals at Nankai University (Tianjin, China). Eight-week-old male Sprague Dawley (SD) rats with a body weight in the range of 180–220 g were selected diabetes induction. Due to its high efficacy and reproducibility, streptozotocin (STZ) is a typically used drug for inducing diabetes in rats. The dose of STZ was 50 mg/kg, and the STZ solution was injected into the left lower abdominal cavity of the rats. After the injection, the blood glucose was measured at 3, 7, 14 and 21 days. When measuring blood glucose at 3 and 7 days, if the blood sugar value is lower than 16.65 mmol/L, an appropriate amount of STZ solution can be injected to ensure that the blood sugar glucose of SD rats is always above 16.65 mmol/L. If the blood glucose of rats was lower than 8.9 mmol/L before induction and higher than 16.65 mmol/L after 21 days of injection, the diabetic rat model was considered successful. The hair on the back of the diabetic rats was shaved, and a circular full-thickness skin wound with a diameter of 10 mm was made on the back of the rat with a sterile scalpel. We used a silicone ring to prevent shrinkage of the wound tissue. The Control (PBS group), rhCol III hydrogel, EVs or rhCol III-EVs hydrogel treatments were applied to the wounds. The wound dressing material was replaced every 3 days. There were five rats in each group. ImageJ (v1.8.0) was used to measure the wound area and calculate the wound healing rate. The wound healing rate was (M_0_−M_1_)/M_0_ × 100%, where M_0_ is the wound area at 0 days and M_1_ is the wound area at 3, 7 and 14 days.

### 4.18. HE Staining

The tissue of the skin wound was fixed with 4% paraformaldehyde, dehydrated and embedded in paraffin. After the sectioned samples were taken out of the refrigerator, they were rewarmed at room temperature for 10 min and rinsed with water for 10 min to fully remove the tissue embedding agent. The slides were stained with hematoxylin, differentiated with hydrochloric acid differentiation solution, stained with eosin, dehydrated in alcohol with a gradient concentration and finally soaked in xylene for transparency. After drying, the film was sealed with neutral gum and photographed with an optical microscope for analysis.

### 4.19. Masson Staining

The sections were stained in Weigert iron hematoxylin staining solution for 7 min. Masson was applied with blue solution for 3 min and returned to blue, rinsed with running water for 5 min and then placed in Ponceau staining solution for 5 min. Then, the weak acid working solution was prepared according to the volume ratio of distilled water: weak acid solution 2:1, and the slides were washed for 1 min. The slides were stained with aniline blue staining solution for 40 s, washed with weak acid working solution for 1 min and soaked in gradient ethanol for dehydration. Xylene was used 3 times to achieve transparency, and the slides were mounted with neutral gum.

### 4.20. Western Blot

The healing tissue of SD rat skin wounds was extracted using RIPA lysate for protein extraction. Extracted proteins were subjected to SDS-PAGE electrophoresis and transfer. The transferred PVDF membrane was sealed with 5% nonfat dry milk for 2 h. After blocking, the corresponding primary and secondary antibodies were incubated. After incubation with the secondary antibody, the PVDF membrane was developed using the ECL Luminescence Kit.

### 4.21. Immunofluorescence Staining for CD31 and α-SMA

The sections were dewaxed into water then placed in EDTA citric acid repair solution for high pressure repair for 5 min. The slides were blocked with goat serum blocking solution for 30 min. The blocking serum was discarded and the mixed primary antibody working solution diluted in PBS was used to incubate overnight at 4 °C. The mixed fluorescent secondary antibody diluted in PBS was dropped on the glass slide and incubated at 37 °C in the dark for 30 min. DAPI staining solution was added dropwise to protect the nucleus from light for 10 min, rinsed with distilled water and mounted with water-soluble mounting tablets.

### 4.22. Immunohistochemical Staining for Ki67 and IL-6

The sections were dewaxed into water and placed in the citric acid repair solution and repaired under high pressure for 5 min. Incubation with 3% hydrogen peroxide was used to reduce endogenous peroxidase activity. The slides were blocked with goat serum blocking solution for 30 min. The primary antibody was added dropwise to the slices and incubated overnight at 4 °C. After the slides were taken out the next day, they were washed 3 times with PBS for 5 min each. The secondary antibody was added dropwise to the sections and incubated at 37 °C for 20 min in the dark, followed by washing with PBS for 5 min each. When the DAB chromogenic solution was used to develop the color of the positive cells, the reaction was terminated by washing with water. After being counterstained with hematoxylin, dehydrated with alcohol and made transparent with xylene, the slides were sealed with neutral glue.

### 4.23. Statistical Analysis

The mean ± standard deviation (SD) was used to represent each group of data and the data was statistically analyzed using GraphPad Prism 7. One-way ANOVA and SNK tests were used to analyze differences in the data. A *p*-value < 0.05 or < 0.01 was considered to indicate a statistical difference between the groups.

## 5. Conclusions

In summary, we prepared a rhCol III hydrogel using a TGase enzymatic cross-linker and successfully loaded it with hUC-MSCs-secreted EVs. The rhCol III-EVs hydrogel achieved local sustained release of EVs and effectively promoted the anti-inflammatory, proliferation, migration and tube formation abilities of wound cells. In addition, it promoted wound healing and tissue regeneration as a dressing on wounds in diabetic rats. Overall, the multifunctional rhCol III-EVs hydrogel could achieve the sustainable release of EVs, accelerate wound healing and provide a novel approach for the treatment of chronic wounds.

## Figures and Tables

**Table 1:** Primers used for qRT-PCR.

Gene	Sequence (5′–3′)
mmu_*Nos2*	F: ATCTTGGAGCGAGTTGTGGATTGTC
	R: TCGTAATGTCCAGGAAGTAGGTGAGG
mmu_*TNFα*	F: ATGTCTCAGCCTCTTCTCATTC
	R: GCTTGTCACTCGAATTTTGAGA
mmu_*Arg1*	F: CATATCTGCCAAAGACATCGTG
	R: GACATCAAAGCTCAGGTGAATC
mmu_*TGFb*	F: CCAGATCCTGTCCAAACTAAGG
	R: CTCTTTAGCATAGTAGTCCGCT
mmu_*GAPDH*	F: ACCCAGAAGACTGTGGATGG
	R: ACACATTGGGGGTAGGAACA

## Data Availability

All data are available in the main text or the Supplementary Information.

## Supplementary Materials

The following supporting information can be downloaded at: https://www.mdpi.com/article/10.3390/ijms23116289/s1.
