# Supplementary material for: Preparation of Recombinant Human Collagen III Protein Hydrogels with Sustained Release of Extracellular Vesicles for Skin Wound Healing
Source: Int J Mol Sci. 2022 Jun 3;23(11):6289. doi: 10.3390/ijms23116289 (PMC9181212; doi:10.3390/ijms23116289)
Supplement: Supplementary file 1 [file ijms-23-06289-s001.zip › ijms-1735167-supplementary.pdf]

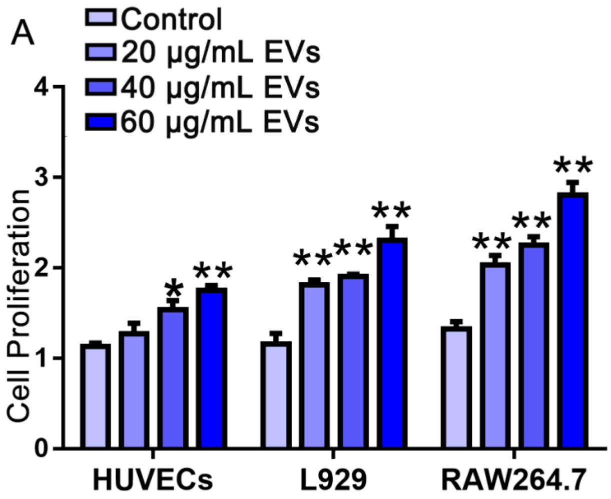

Figure S1 (A) The proliferation of HUVECs, L929 cells and RAW264.7 cells in different groups (n=3, *P-value<0.05 vs Control group, **P-value<0.01 vs Control group).
